# Prevalence and influence factors of vitamin A deficiency of Chinese pregnant women

**DOI:** 10.1186/s12937-016-0131-7

**Published:** 2016-01-27

**Authors:** Chun Yang, Jing Chen, Zhen Liu, Chunfeng Yun, Jianhua Piao, Xiaoguang Yang

**Affiliations:** Key Laboratory of Trace Element Nutrition, National Health and Family Planning Commission of the people’s Republic of China, Department of Trace Element Nutrition, National Institute for Nutrition and Health, Chinese Center for Disease Control and Prevention, Room 236, Nanwei Road No.29, Xicheng District Beijing, China 100050

**Keywords:** Vitamin A deficiency, Chinese pregnant women, Influence factors

## Abstract

**Background:**

Vitamin A plays an important role in the periods of rapid cellular growth and differentiation, especially during pregnancy, which is supplied by the mother to the fetus. The aim of this study is to assess the prevalence and potential influence factors of prenatal VAD of Chinese pregnant women.

**Methods:**

China National Nutrition and Health Survey 2010–2013(CHNNS2010–2013) is a nationally representative cross-sectional study. It involved the random selection of 150 districts (urban) or counties (rural). Each site randomly selected 30 pregnant women. Because volume of blood and incomplete data was taken into consideration,the final sample was formed by 1209 participants. Serum retinol concentrations were measured by high performance liquid chromatography. Characteristics of the pregnant women were collected by a questionnaire. Comparing retinol level across categories of independent variables was tested by the Mann-Whitney *U* test. Logistic and linear regression analyses were used to identify influence factors of Chinese pregnant women.

**Results:**

The mean serum retinol level of the pregnant women was 1.63 μmol/L (95 % CI 1.60–1.67) and 64[5.3 % (95 % CI 4.03–6.56)] had VAD. The odds of VAD were significantly higher among the pregnant women in the poor rural areas and without college or university education and low- income. Pregnant women in the second and third trimester had 2.40 (95 % CI 1.05–5.46) and 2.82 (95 % CI 1.34–5.93) times increased odds of VAD compared with those in the first trimester respectively. Pregnant women of drinker had 3.10(1.65–5.81) times increased odds of VAD compared with those no drinker. Pregnant smokers had 5.68 (95 % CI 2.23–14.49) times higher odds of VAD compared with pregnant with non-smoker without passive smoking.

**Conclusions:**

VAD is of mild public-health issue in Chinese pregnant women. Such as : in the poor rural areas and without received college or university education and low- income and advanced gestational age and unhealthy lifestyles of pregnant women such as smoking and drinking. These were pertinent influence factors of VAD.

## Background

Vitamin A is an essential nutrient needed in small amounts for the normal functioning of the visual system, and maintenance of cell function for growth, epithelial integrity, red blood cell production, immunity and reproduction [1]. The World Health Organization (WHO) defines Vitamin A deficiency (VAD) as tissue concentrations of vitamin A (VA) low enough to have adverse health consequences, even if there is no evidence of clinical deficiency [2]. VAD is recognized as a major public-health nutrition issue in the developing countries [3]. VAD has been more damaging and predominantly effect to pregnant women, indeed by WHO estimates, 19 million pregnant women are thought to be affected by this nutritional deficiency [1].

The majority of studies focus on VAD of pregnant women, it found vitamin A deficiency was strongly associated with pregnancy and birth outcomes. They found antenatal vitamin A supplementation decreased anemia [4, 5], preterm delivery [5, 6], intrauterine growth retardation [6], low birth weight [4], deformity [7], preeclampsia/eclampsia [8, 9], poor infant growth [4], neonatal and infant mortality [4, 10], and maternal mortality [10, 11]. Vitamin A status is assessed by the serum concentration of retinol, but there was little about its value as a marker of vitamin A status during pregnancy in China. A previous study [12] which based on small sample size found that Chinese pregnant women’s vitamin A concentration was 1.13 ± 0.37 μmol/L, and 24 of 143 pregnant women (16.80 %) was vitamin A deficiency, while another 52 pregnant women (36.4 %) was marginal vitamin A deficiency. Our study based on large sample of Chinese pregnant women and aimed to assess the prevalence and potential influence factors of VAD of Chinese pregnant women.

## Methods

### Study design and participants

Data for the present analysis were obtained from the China National Nutrition and Health Survey 2010–2013(CHNNS2010–2013). This survey is a nationally representative cross-sectional study that has been conducted by Chinese Center for Disease Control and Prevention to assess the health and nutrition of Chinese civilians. It covered all 31 provinces, autonomous regions, and municipalities directly under the central government throughout China (except Taiwan, Hong Kong, and Macao). A stratified multistage probability sampling design was used for the selection of participants. The country was divided into four strata by their economic characteristics and social development. They were large cities, small to medium cities, general rural areas and poor rural areas. It involved the random selection of 150 districts (urban) or counties (rural). The pregnant women serum samples were collected in 2013–2014. All the participants were given a urine human chorionic gonadotropin test. We excluded participants with a history of kidney disease or chronic liver disease. Each site randomly selected 30 pregnant women. The technical problems in the volume of blood and incomplete data were taken into consideration,the final sample was formed by 1209 participants whose serum concentrations of retinols were present in the data bank.

### Ethical considerations

The study was conducted according to the guidelines laid down in the Declaration of Helsinki and all procedures involving human subjects were ethically approved by the Ethics committee of Institute for Nutrition and Health, Chinese Center for Disease Control and Prevention. Those who voluntarily decided to participate signed an informed consent form.

### Data collection

A series of questionnaires was used for CHNNS2010–2013. The questionnaires were completed by centralized trained investigators. The questionnaires included questions regarding the pregnant such as:age; gestational age; parity; whether use the vitamin A supplements; pregnant women drinkers defined as pregnant women who drink alcohol during the past 12 months; education levels of the pregnant ; whether received college or university education; annual income of the pregnant women RMB(yuan) was divided into four levels: <10,000,10,000–20,000,20,000–30,000, > = 30,000; smoking habit was divided into three categories: non-smoker without passive smoking, non-smoker with passive smoking,smokers. All the participants were asked to show the medical report finished by licensed doctor and the medical report was must within half years before pregnancy.

### Blood sample collection,serum extraction,and laboratory analysis

Fasting blood samples were obtained by venipuncture from the antecubital vein in the morning of the next day after questioning, centrifuged within 0.5–1 h after collecting. Serum was extracted and aliquoted immediately into screw-top vials. The samples were transported in icebox, protected from direct light, and stored at −80 °C until analyzed.

Serum retinol concentrations were used high performance liquid chromatography (Waters 2487UV detector USA) [13]. Retinol was extracted with hexane after deproteinization with ethanol containing retinyl acetate as the internal standard and evaporated to dryness with nitrogen gas. The residual materials were dissolved in 0.2 mL ethanol. The mobile phase was a methanol: DH2O mixture (96:4). A portion (10 μL) of the sample was injected into a column (3.9 150 mm; Symmetry Shield RPl8 Waters Breeze, Milford, MA, USA) installed with a high-performance liquid chromatographic apparatus(Waters 1525 Binary HPLC Pump, Waters Breeze). All procedures were performed in a dark room to protect the serum from light. The concentration of retinol was determined with a spectrophotometer(Waters 2487 Dual Absorbance Detector, Waters Breeze) at 325 nm. Duplicate analyses were performed on one-tenth of the samples and the estimated variability was 0.02 μmol/L. The experienced examiners measured all biochemical indices. VAD (serum retinol <0.7 μmol/L) and marginal VAD (serum retinol 0.7 to 1.05 μmol/L) were defined according to the recommendation of WHO [1]. Hemoglobin concentrations were determined with the cyanmethemoglobin method and anemia was defined as <110 g/L [14].

### Statistical analyses

SAS version 9.2 (SAS Institute, Inc, Cary, North Carolina) was used for data-entry, screening, and analysis. The Kolmogorov-Smirnov test was used for analysis the normality of the distribution of serum retinol level of each category and found them did not obeyed normal distribution. Descriptive analysis was done using mean, 95 % confidence interval, frequency, and percentage. Comparing retinol level across categories of independent variables was used the Kruskal-Wallis test followed by the Mann-Whitney *U* test. Logistic and linear regression analyses were used in controlling potential confounders. Independent variables significantly (*p* < 0.05) associated with the dependent variable in simple regression models were exported to multiple regression models for adjustment. The major assumptions of logistic regression analysis (absence of influential cases, multicollinearity and interaction among independent variables) and linear regression analysis (normally-distributed error terms, linear relation between dependent and independent variables, homoscedasticity, and absence of multicollinearity) were checked to be satisfied. The fitness of logistic and linear regression models were assessed using Hosmer-Lemeshow statistic and adjusted r-squared values respectively.

## Results

### Vitamin A status of Chinese pregnant women

The mean serum retinol level was 1.63 μmol/L (95 % CI 1.60–1.67), and 64[5.3 % (95 % CI 4.03–6.56)] of the participants had VAD, additional 137 [11.3 % (95 % CI 9.54–13.12)] participants had marginal VA status, normal was 1008[83.4 % (95 % CI 82.8–85.47)].

### Comparison of characteristics of Chinese pregnant women

The retinol concentrations for pregnant women age groups were significant difference (*F* = 6.78, *p* = 0.03). Across the levels of pregnant women education had significant difference (*F* = 6.24, *P* = 0.01). With the increase of income the retinol levels of pregnant increased,the global difference across the categories was significant difference (*F* = 12.63, *p* = 0.01).As aggravation of smoking the retinol levels of pregnant decreased,the global difference across the categories was significant (*F* = 5.92, *p* = 0.00). The retinol level in the first trimester was significantly higher than the corresponding values for the third and second trimesters (*F* = 25.47, *p* = 0.00). Significant difference in retinol concentrations was witnessed across parity of 0, 1, and 2 or more(*F* = 7.66, *p* = 0.02); The difference between pregnant women whether was anemia was significant (*F* = 10.28, *p* = 0.00). Pregnant women with vitamin A supplements compared with without vitamin A supplements was significant difference (*F* = 8.67, *p* = 0.00). The group of drinker between yes or no was significant difference (*F* = 21.55, *P* = 0.00). The retinol concentrations for women between different regions had no significant (Table 1).

**Table 1:** Comparison of characteristics of Chinese pregnant women

Variable	N	Mean(95 % CI) μmol/L	F	*P*
Age			6.78	0.03
15–25	490	1.68(1.63–1.74)		
25–35	663	1.65(1.47– 1.84)		
35–45	56	1.57(1.51 –1.62)*		
Region strata			6.62	0.08
Large cities	287	1.63(1.56 –1.71)		
Small to medium cities	360	1.67(1.60– 1.74)		
General rural areas	291	1.65(1.57–1.73)		
Poor rural areas	271	1.59(1.49–1.68)		
College or university			6.24	0.01
No	832	1.60(1.56 –1.65)		
Yes	377	1.70(1.63–1.76)		
Income			12.63	0.01
< 10,000	199	1.58(1.52–1.63)*		
10,000–20,000	339	1.63(1.55– 1.71)		
20,000–30,000	486	1.64(1.54 –1.75)		
> =30,000	185	1.77(1.68–1.86)*		
Drinkers			21.55	0.00
No	1071	1.65(1.61–1.69)		
Yes	138	1.52(1.40–1.64)		
Smoking			5.92	0.00
Non-smoker without passive smoking	553	1.67(1.61–1.72)		
Non-smoker with passive smoking	625	1.62(1.57–1.67)		
Smokers	31	1.26(0.98–1.53)*		
Gestational age			25.47	0.00
First trimester	471	1.74(1.68–1.80)*		
Second trimester	292	1.64(1.56–1.71)		
Third trimester	446	1.52(1.46–1.58)*		
Parity			7.66	0.02
0	877	1.70(1.62–1.79)		
1	299	1.62(1.58–1.66)		
2+	33	1.36(1.14–1.59)*		
Anemia			10.28	0.00
No	916	1.67(1.63–1.71)		
Yes	293	1.52(1.45–1.60)		
Vitamin A supplement			8.67	0.00
No	1104	1.62(1.58–1.66)		
Yes	105	1.80(1.67–1.91)		

*compared other group *p* < 0.05 was significant difference

### Linear regression modelling

In the linear regression model, the serum retinol level (μmol/L) was explained by 5 independent variables (Table 2). Unit increment in age (in years) and gestational age (in months) were associated with 0.01and 0.08 μmol/L decline in serum retinol level respectively. According increment levels of region strata and anemia were associated with 0.05 and 0.11 μmol/L decline in serum retinol level respectively. On the contrary, a unit increment in level of income was associated with 0.05 μmol/L rise in retinol concentration (Table 2).

**Table 2:** Linear regression output on the predictors of serum retinol concentrations (μmol/L) among pregnant women in China

Independent variable	Unstandardized coefficient	t-statistic	*P* value
Constant	1.61		
age (years)	−0.01	−2.34	0.02
Gestational age (months)	−0.08	−3.68	0.00
Region strata^a^	−0.05	−2.42	0.02
Anemia(0 = yes 1 = no)	−0.11	−2.54	0.01
Income+	0.05	2.53	0.01

^a^:1 = large cities 2 = small to medium cities 3 = general rural areas 4 = poor rural areas

+:1:<10,000;2:10,000–20,000;3:20,000–30,000;4:≥30,000

### Logistic regression modelling

Logistic regression analyses showed that pregnant women in the poor rural areas had 4.73 (2.67–8.41) times increased odds of VAD compared with those in the large cities. Pregnant women without college or university education had 3.17 times (95 % CI 1.51–6.67) higher odds of VAD compared with pregnant with college or university education. Pregnant women’s income at 20,000–30,000, 10,000–20,000, <10,000 had 3.64 (1.01–13.16), 4.33 (1.18–15.90) and 4.91 (1.31–18.46) times higher odds of VAD compared with pregnant women income beyond 30,000. Across the group of drinkers between yes or no with pregnant women found drinkers had 3.10 (1.65–5.81) times increased odds of VAD compared with those no drinkers. Pregnant smokers had 5.68 times (95 % CI 2.23–14.49) higher odds of VAD compared with pregnant with non-smoker without passive smoking. Pregnant women in the second and third trimester had 2.40 (95 % CI 1.05–5.46) and 2.82 (95 % CI 1.34–5.93) times increased odds of VAD compared with those in the first trimester respectively (Table 3).

**Table 3:** Vitamin A deficiency and potential influence factors among pregnant women in China

Variable	VAD+	VAD–	Crude OR (95 % CI)	Adjusted OR (95 % CI)
Region strata				
Large cities	11	276	1	1
Small to medium cities	13	347	0. 62(0.24–1.62)	–
General rural areas	7	284	0.94(0.42–2.13)	–
Poor rural areas	33	238	3.48(1.72–7.03)^a^	4.73(2.67–8.41)^a^
College or university				
No	52	780	2.03(1.07–3.85)^a^	3.17 (1.51–6.67)^a^
Yes	12	365	1	1
Income				
< 10,000	14	185	4.60(1.30–16.24)^a^	4.91 (1.31–18.46)^a^
10,000–20,000	22	317	4.21(1.24–14.26)^a^	4.33 (1.18–15.90)^a^
20,000–30,000	25	461	3.29(0.98–11.03)^a^	3.64 (1.01–13.16)^a^
> =30,000	3	182	1	1
Drinking				
Yes	20	118	4.00(2.26–6.94)^a^	3.10 (1.65–5.81)^a^
No	44	1027	1	1
Smoking				
Non-smoker without passive smoking	24	529	1	1
Non-smoker with passive smoking	31	594	1.15(0.67–1.99)	–
Smokers	9	22	9.02(3.75–21.67)^a^	5.68 (2.23–14.49)^a^
Gestational age				
First trimester	10	461	1	1
Second trimester	18	274	3.03(1.38–6.66)^a^	2.40(1.05–5.46)^a^
Third trimester	36	410	4.05(1.98–8.26)^a^	2.82(1.34–5.93)^a^

^a^Statistically significant at 0.05 level of significance

## Discussion

Our study first reported about the vitamin A nutritional status in a large group of Chinese pregnant women. The level of vitamin A was 1.63 μmol/L and VAD was 5.3 %. According to WHO, the public-health significance of VAD is considered to be of mild degree when the prevalence of low serum retinol (<0.70 μmol/L) in pregnant women between 2 and 10 % from the reference value [1]. So VAD is of mild public-health issue in Chinese pregnant women. Compared with other studies in region of developing countries that VAD was found among 20 %. (in Egypt), 15.8 % (in Nigeria) and 18.8 % (in Bangladesh) of pregnant women [15–17]. The lower frequency of VAD in the present study may be explained by improving the socioeconomic level.

In the current study, pregnant women age and VA status were negatively associated and the level of vitamin A decreased with age. The same result found in India and Ethiopia [18, 19]. The reason might be that pregnant women’s physiological state changed with the increase of age and decreased maternal store of retinol. Present study found multiple reproductive was lower the Chinese pregnant women VA level. Consistent with previous study in the other developing country [18]. It is consistent with the understanding that repeated reproductive cycles deplete maternal store of nutrients [20]. The present study showed that retinol concentrations of anemia and pregnant women with vitamin A supplements were higher than no anemia and pregnant women without vitamin A supplements. Anemia and VA status were positively associated in our study. Vitamin A plays an important role in hematopoiesis, and anemia always caused VAD [21]. Vitamin A supplementation during pregnancy was found to increase the hemoglobin concentrations and reduced the VAD occurred [22].

In our study, gestational trimester and VA status were negatively associated in Chinese pregnant. The same result was also reported in other countries studies; such as in Guinea [23] and Ethiopia [18]. In our study, serum retinol significantly declined by about 13 % from the first (1.74 μmol/L) to the third trimester (1.52 μmol/L). Parallel finding were reported that serum retinol was considerably lower, ~0.10 mmol/L or 10 % from wk 20 onwards, compared with wk 7–16 of pregnancy. This may reflect a true reduction in vitamin A stores with increasing gestation, as proposed by a large trial of weekly vitamin A supplementation in Bangladesh [24]. However, a decrease in serum retinol during pregnancy due to the increased plasma volume has been reported [25]. We also founded that pregnant women in the second and third trimester had 2.40 (95 % CI 1.05–5.46) and 2.82 (95 % CI 1.34–5.93) times increased odds of VAD compared with those in the first trimester respectively used the cutoff value(serum retinol <0.7 μmol/L). If appropriate cutoff values indicating deficiency need to be established, further study need for having trimester-specific cutoff points for defining prenatal VAD.

The nutritional status of a population to a great extent depends on socioeconomic factors. In the previous study found that socioeconomic status was a vital causal factor for VAD [16]. We divided into four regions according to the economic development of China areas, VA status and levels of region strata were negatively associated in our study, although the retinol concentrations for pregnant women between different regions had no significant difference, pregnant women in the poor rural areas had 4.73 (2.67–8.41) times increased odds of VAD compared with those in the large cities. And VA status and the level of education were positively associated and increased with Chinese pregnant women income. Compared with higher income, the lower income was easily to vulnerable VAD in Chinese pregnant women. The reason maybe that population was easily to access to foods rich in preformed vitamin A. Educational status of the women in this study correlated positively with their serum retinol levels. Also, pregnant women without college or university education had 3.17 times (95 % CI 1.51–6.67) higher odds of VAD compared with pregnant women with college or university education. The reason may be that higher educational status in pregnant women leads to improved income, health and nutrition knowledge, food habits, nutrient intake, self–concept and health seeking behavior [26].

Unhealthy lifestyles of pregnant women such as smoking and drinking always bring adversely affect. In present study founded that the retinol concentrations of smoker and drinker of pregnant women were lower than non-smoker and non- drinker of pregnant. And also pregnant women of drinker had 3.10 (1.65–5.81) times increased odds of VAD compared with those no drinker, pregnant smokers had 5.68 times (95 % CI 2.23–14.49) higher odds of VAD compared to pregnant with non-smoker without passive smoking.so tobacco and alcohol used during pregnancy prone to occur VAD. Tobacco use was linked to pregnancy complications, low-birth weight infants, still birth and sudden infant death [27, 28]. Alcohol consumption of pregnant women increased the risk of a child adversely affected such as fetal alcohol spectrum disorders and birth defects even developmental disorders [29–31].

One major limitation of the present study is a cross-sectional study. It is also lack of assessment of the participants’ physical behavior condition which influences vitamin A status of Chinese pregnant women. More studies are needed to understand the influence factors of vitamin A status of Chinese pregnant women.

## Conclusions

In summary, the current study reported the prevalence of vitamin A among Chinese pregnant women and the prevalence of VAD was 5.3 %, which was a mild public-health issue in China. In the poor rural areas and without college or university education and low- income and advanced gestational age were pertinent risk factors of VAD. Unhealthy lifestyles of pregnant women such as smoking and drinking were also pertinent influence factors of VAD. So in the future health public policy on vitamin A should be paid more attention on pregnant women with pertinent influence factors of VAD, accordingly, eliminate VAD improve the nutritional status of Chinese pregnant women.
